# Sex differences in risk factors for end‐stage kidney disease and death in type 2 diabetes: A retrospective cohort study

**DOI:** 10.1111/1753-0407.13367

**Published:** 2023-02-13

**Authors:** Megumi Oshima, Yasunori Iwata, Tadashi Toyama, Shinji Kitajima, Akinori Hara, Norihiko Sakai, Miho Shimizu, Kengo Furuichi, Masakazu Haneda, Tetsuya Babazono, Hiroki Yokoyama, Kunitoshi Iseki, Shinichi Araki, Toshiharu Ninomiya, Shigeko Hara, Yoshiki Suzuki, Masayuki Iwano, Eiji Kusano, Tatsumi Moriya, Hiroaki Satoh, Hiroyuki Nakamura, Hirofumi Makino, Takashi Wada

**Affiliations:** ^1^ Department of Nephrology and Laboratory Medicine Kanazawa University Kanazawa Japan; ^2^ Innovative Clinical Research Center Kanazawa University Kanazawa Japan; ^3^ Department of Environmental and Preventive Medicine Kanazawa University Kanazawa Japan; ^4^ Department of Nephrology Kanazawa Medical University Uchinada Japan; ^5^ Department of Medicine Asahikawa Medical University Asahikawa Japan; ^6^ Division of Diabetology and Metabolism, Department of Internal Medicine Tokyo Women's Medical University School of Medicine Tokyo Japan; ^7^ Jiyugaoka Medical Clinic, Internal Medicine Obihiro Japan; ^8^ Okinawa Heart and Renal Association Naha Japan; ^9^ Division of Nephrology, Department of Internal Medicine Wakayama Medical University Wakayama Japan; ^10^ Department of Epidemiology and Public Health Graduate School of Medical Sciences, Kyushu University Fukuoka Japan; ^11^ Center of Health Management, Toranomon Hospital Tokyo Japan; ^12^ Okinaka Memorial Institute for Medical Research Tokyo Japan; ^13^ Niigata University Medical and Dental Hospital Niigata Japan; ^14^ Department of Nephrology Faculty of Medical Sciences, University of Fukui Fukui Japan; ^15^ Division of Nephrology, Department of Internal Medicine Jichi Medical University Tochigi Japan; ^16^ Health Care Center, Kitasato University Sagamihara Japan; ^17^ Department of Diabetes and Endocrinology Juntendo University Urayasu Hospital Chiba Japan; ^18^ Okayama University Okayama Japan

**Keywords:** albuminuria, death, end‐stage kidney disease, sex difference, type 2 diabetes, 性别差异, 2型糖尿病, 终末期肾脏疾病, 白蛋白尿, 死亡

## Abstract

**Background:**

This study investigated the sex differences in the risk of end‐stage kidney disease (ESKD) and mortality, as well as the effect modification of sex on associated factors in patients with type 2 diabetes.

**Methods:**

This multicenter observational cohort study included 4328 patients with type 2 diabetes. Hazard ratios (HRs) with 95% confidence intervals (CIs) of sex for ESKD and death were estimated using Cox proportional regression with adjustment for baseline covariates. For assessing risk modification, HRs and incidence rates for ESKD and death were compared between sexes across patient characteristics using Cox proportional and Poisson regression models.

**Results:**

During a median follow‐up of 7 years, 276 patients (70% men) developed ESKD, and 241 patients (68% men) died. Men had higher risks of ESKD (HR 1.34; 95% CI 1.02–1.75; *p* = .034) and death (HR 1.64; 95% CI 1.24–2.16; *p* = .001) versus women after adjusting for multiple covariates. Among patients with microalbuminuria, men had a substantially higher risk of ESKD versus women, compared to those with normo‐ and macroalbuminuria (*p* for interaction .04). Incidence rates were also increased in men versus women with albuminuria of around 300 mg/g. No differences were detected in the association of sex and death across baseline patient subgroups.

**Conclusions:**

In type 2 diabetes, men had an increased risk of ESKD and death versus women. Moderately increased albuminuria was strongly associated with sex difference in developing ESKD.

## Graphical abstract

**Highlights**

- Sex differences are observed in the progression of chronic kidney disease; however, it is uncertain whether sex is associated with the risk of kidney failure in type 2 diabetes.
- In this multicenter cohort study, men had a higher risk of end‐stage kidney disease (ESKD) than women, and moderately increased albuminuria was strongly associated with sex difference in developing ESKD in patients with type 2 diabetes.
- Sex differences may affect the risk of kidney disease progression because of type 2 diabetes.

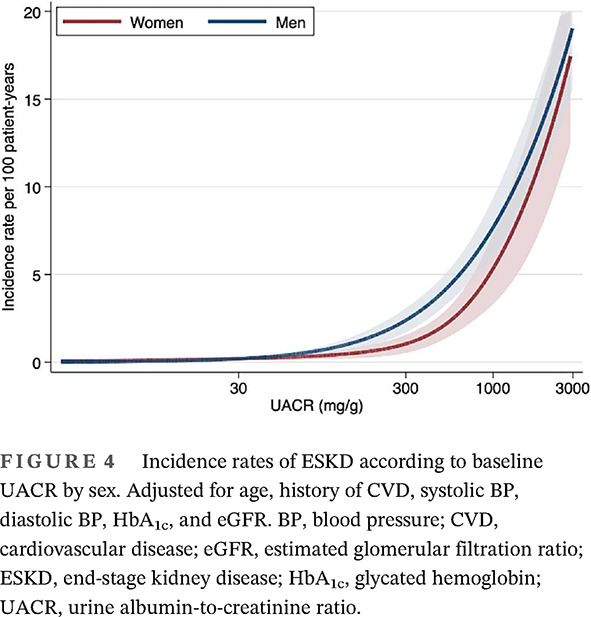

## 摘要

**背景:** 本研究调查了2型糖尿病患者中性别差异对终末期肾脏疾病(ESKD)和死亡风险的影响, 以及性别对相关因素的调节效应。

**方法:** 这项多中心观察性队列研究包括4,328名2型糖尿病患者。使用Cox比例风险回归模型调整基线协变量, 估计性别对ESKD和死亡的风险比(HR)和95%置信区间(CI)。为评估风险调节, 使用Cox比例风险回归和泊松回归模型比较性别在患者特征上对ESKD和死亡的风险比和发生率。

**结果:** 在中位随访7年期间, 276名患者(男性70%)出现了ESKD, 而241名患者(男性68%)死亡。在调整多个协变量后, 男性相比女性患ESKD(HR 1.34; 95% CI 1.02, 1.75; p = 0.034)和死亡(HR 1.64; 95% CI 1.24, 2.16; p = 0.001)的风险更高。在微量白蛋白尿患者中, 相比于无蛋白尿和高度蛋白尿的患者, 男性相比女性患ESKD的风险要高得多(交互作用p值为0.04)。在约为300 mg/g的蛋白尿患者中, 男性的发病率也比女性高。在基线患者亚组中, 没有发现性别和死亡的关联差异。

结论:在2型糖尿病患者中, 男性相比女性患ESKD和死亡的风险更高。中度增加的蛋白尿与性别在发展ESKD方面的差异密切相关。

## 1 INTRODUCTION

Diabetic kidney disease (DKD) develops in ~40% of people with type 2 diabetes, and it remains a leading cause of end‐stage kidney disease (ESKD) and early mortality worldwide.^1^, ^2^ Various explanatory factors have been associated with a greater risk of the onset and progression of kidney disease in patients with type 2 diabetes.^3^ These factors can be used for early risk stratification and targeted intervention to prevent adverse kidney outcomes.

The cumulative incidence and risk of kidney replacement therapy have been reported to be higher in men than women with chronic kidney disease (CKD).^4^ A study of patients with type 2 diabetes also demonstrated that men had a faster decline in kidney function versus women,^5^ suggesting that sex differences may exist in the progression of CKD. However, it is uncertain whether sex is associated with the risk of ESKD and which factors affect the associations between sex and kidney outcomes in patients with type 2 diabetes. To address this knowledge gap, this study investigated the sex differences in kidney prognosis and the effect modification of sex on its associated factors in patients with type 2 diabetes using data from a multicenter cohort study with long‐term follow‐up.

## 2 MATERIALS AND METHODS

### 2.1 Patients and study design

The observational cohort used in this study included 4328 patients with type 2 diabetes treated across 10 centers in Japan between 1985 and 2011. Patients were diagnosed with type 2 diabetes according to the criteria of the Japan Diabetes Society (JDS).^6^ The exclusion criteria were as follows: age <18 years, type 1 or secondary diabetes, kidney transplantation, maintenance dialysis, missing values of baseline covariates (including urine albumin‐to‐creatinine ratio [UACR], estimated glomerular filtration rate [eGFR], glycated hemoglobin [HbA_1c_], and systolic blood pressure [BP]), and refusal to provide informed consent. A detailed description of the study design was previously reported.^7^ Other background therapies for glycemic management and control of cardiovascular risk factors were used according to the recommendation of the local guidelines. Patients were followed up until the onset of the first study outcome or the end of follow‐up in October 2011. All participants provided written informed consent and study protocols were approved by the local institutional ethics committees at each site.

### 2.2 Baseline and follow‐up variables

A history of cardiovascular disease (CVD) was defined as having at least one of following; coronary heart disease, stroke, cerebral hemorrhage, heart failure, or arteriosclerosis obliterans. BP was measured in the sitting position. HbA_1c_ was measured according to the standards of the JDS, using nonfasting blood samples, and converted into HbA_1c_ via the National Glycohemoglobin Standardization Program (JDS + 0.4) method for the analysis. Serum creatinine level was measured using an enzymatic method, and eGFR was calculated using the equation proposed by the Japanese Society of Nephrology.^8^ Urine albumin and creatinine levels were measured using a turbidimetric immunoassay and an enzymatic method applied on spot urine samples.

### 2.3 Study outcome

The primary outcome was the occurrence of ESKD, defined as the need for renal replacement therapy or having an eGFR of <15 mL min^−1^ 1.73 m^−2^. The secondary outcome was death.

### 2.4 Statistical analyses

Baseline patient characteristics were summarized and stratified according to sex. Continuous variables are presented as mean with SD for variables with approximately symmetrical distributions. The results for variables with skewed distributions are shown as the median and interquartile range (IQR); these were transformed into natural logarithms before analysis. Categorical variables are reported as percentages.

Cox proportional regression was performed to estimate hazard ratios (HRs) with 95% confidence intervals (CIs) of sex for ESKD. The models were adjusted for baseline covariates including age, history of CVD, systolic and diastolic BP, HbA_1c_, eGFR, and log‐transformed albuminuria and stratified by institutions. Additionally, Kaplan–Meier curves were determined among men and women. In the sensitivity analysis, we repeated the analysis after including death as a competing risk.

For assessing potential effect modification by sex, we evaluated the association between sex and ESKD according to baseline covariates, including age (< 65 or ≥ 65 years), sex, history of CVD, systolic BP (<140 or ≥140 mm Hg), HbA_1c_ (<7.0 or ≥7.0% [<53 or ≥53 mmol/mol]), eGFR (< 60 or ≥60 mL min^−1^ 1.73 m^−2^), and albuminuria (<30, 30–300, or >300 mg/g). The interaction was assessed by adding sex with subgroup interaction terms to the models. Further analyses were performed for the variables indicating effect modification by sex. Incidence rates (per 100 patient‐years of follow‐up) of ESKD were estimated according to the levels of variables using restricted cubic splines via Poisson regression models.

To address the association of sex with eGFR and UACR trajectories, least‐squares mean changes from baseline in eGFR and UACR over time were analyzed by sex using linear mixed‐effects models with restricted maximum likelihood‐based repeated measures. The models included the fixed effects of measurement timepoint and continuous fixed covariates of baseline value and baseline value‐by‐time point interaction. An unstructured covariance structure was used to model within‐patient errors. We also evaluated the association of sex with three phenotypes of clinical course of DKD using Cox proportional or logistic regression as appropriate: (a) progression of albuminuria defined as the development of macroalbuminuria (UACR ≥300 mg/g) from normo‐ or microalbuminuria (<300 mg/g); (b) regression of albuminuria defined as a transition from macroalbuminuria to normo‐ or microalbuminuria or from microalbuminuria (≥ 30 mg/g) to normoalbuminuria (<30 mg/g); and (c) rapid eGFR decline of ≥5 mL min^−1^ 1.73 m^−2^ year^−1^ during follow‐up. These models were adjusted for baseline covariates described previously. Statistical significance was set at *p* < .05, and all analyses were performed using Stata version 16.

## 3 RESULTS

### 3.1 Patient characteristics

Among 4328 patients, 61% (2635 patients) were men. The study cohort had a mean age of 60 years (SD 12), mean HbA_1c_ of 7.6% (SD 1.7) (60 mmol/mol [19]), mean eGFR of 76 mL min^−1^ 1.73 m^−2^ (SD 24), and median UACR of 25 mg/g (IQR 9–66) at baseline (Table 1). Men were more likely to be younger and have history of CVD, lower levels of systolic BP and HbA_1c_, and higher levels of UACR than women. Men and women had a similar mean eGFR at baseline. Among patients whom the year of registration was recorded, similar distributions were observed between men and women, irrespective of the period of registration (Table S1).

**TABLE 1:** Baseline characteristics of patients by sex.

	Total (*n* = 4328)	Men (*n* = 2635)	Women (*n* = 1693)	*P* value
Age, years	60 (12)	59 (11)	62 (12)	<0.001
History of CVD, *n* (%)	309 (7)	215 (8)	94 (6)	0.001
Systolic BP, mm Hg	131 (19)	130 (18)	132 (19)	0.005
Diastolic BP, mm Hg	74 (18)	75 (12)	74 (25)	0.10
HbA_1c_, %	7.6 (1.7)	7.5 (1.7)	7.8 (1.7)	<0.001
HbA_1c_, mmol/ml	60 (19)	58 (19)	62 (19)	<0.001
eGFR, ml min^−1^ 1.73 m^−2^	76 (24)	76 (24)	76 (25)	0.90
eGFR, *n* (%)				0.24
≥90	1132 (26)	675 (26)	457 (27)	
60 ≤ 90	2124 (49)	1329 (50)	795 (47)	
45 ≤ 60	682 (16)	406 (15)	276 (16)	
30 ≤ 45	267 (6)	154 (6)	113 (7)	
<30	123 (3)	71 (3)	52 (3)	
UACR, mg/g	25 (9, 66)	19 (8, 74)	18 (9, 58)	0.006
UACR, *n* (%)				0.001
<30 mg/g	2679 (62)	1581 (60)	1098 (65)	
30 ≤ 300 mg/g	115 (26)	693 (26)	422 (25)	
≥300 mg/g	534 (12)	361 (14)	173 (10)	

*Note*: Variables were demonstrated as mean (standard deviation) or median (first quartile, third quartile).

Abbreviations: BP, blood pressure; CVD, cardiovascular disease; eGFR, estimated glomerular filtration ratio; HbA_1c_, glycated hemoglobin; UACR, urine albumin‐to‐creatinine ratio.

### 3.2 Association between sex and ESKD

**FIGURE 1:**
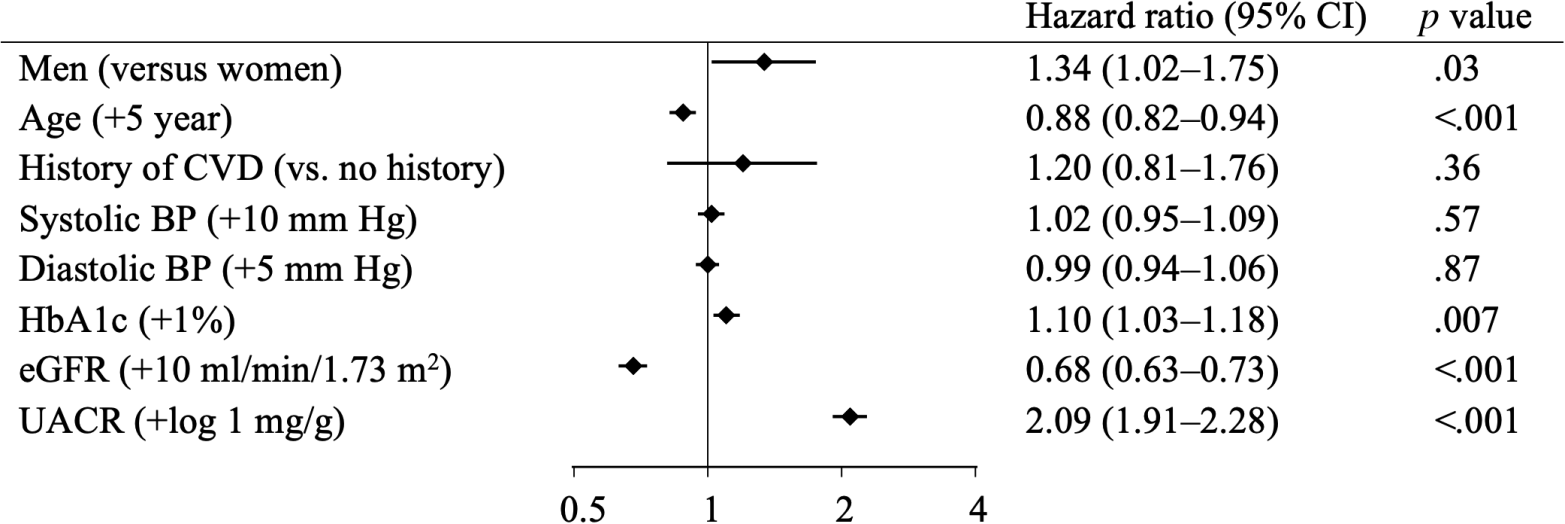
Hazard ratios for the association between baseline covariates and ESKD. Adjusted for age, history of CVD, systolic BP, diastolic BP, HbA_1c_, eGFR, and log‐transformed UACR and stratified by institutions. BP, blood pressure; CI, confidence interval; CVD, cardiovascular disease; eGFR, estimated glomerular filtration ratio; ESKD, end‐stage kidney disease; HbA_1c_, glycated hemoglobin; UACR, urine albumin‐to‐creatinine ratio.

**FIGURE 2:**
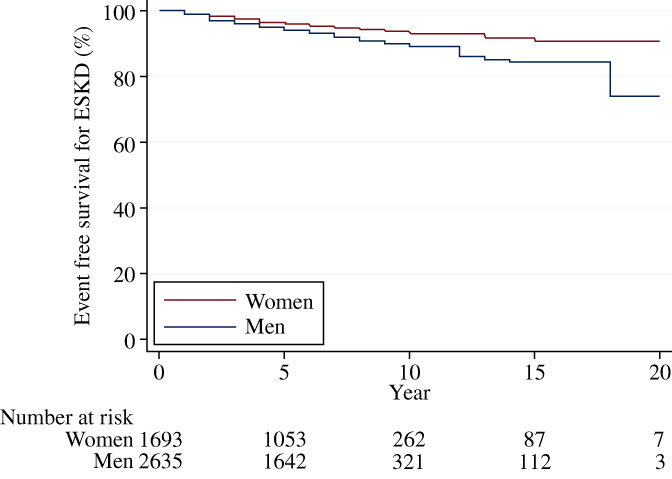
Kaplan–Meier curves for ESKD in women and men. ESKD, end‐stage kidney disease.

Over a median follow‐up of 7 years (IQR 4–8), 276 (6.4%) patients (70% [*n* = 194] men) had ESKD and 241 (5.6%) patients (68% [*n* = 164] men) died. More men reached ESKD than women (11.7 vs. 7.4 patients per 1000 patient‐years). Men were associated with a higher risk for ESKD (HR 1.34; 95% CI 1.02–1.75; *p* = .03) compared to women after adjusting for multiple covariates (Figure 1). Older age and higher eGFR levels were also associated with a lower risk for ESKD, whereas higher levels of HbA_1c_ and UACR were associated with a higher risk for ESKD. A similar relationship between sex and ESKD was observed in the Kaplan–Meier curves (Figure 2) and after including death as a competing risk (Table S2). Additional analysis revealed that men were also associated with an increased risk for death compared to women (HR 1.64; 95% CI 1.24–2.16; *p* = .001) (Figures S1 and S2).

### 3.3 Sex differences in risk factors for ESKD

**FIGURE 3:**
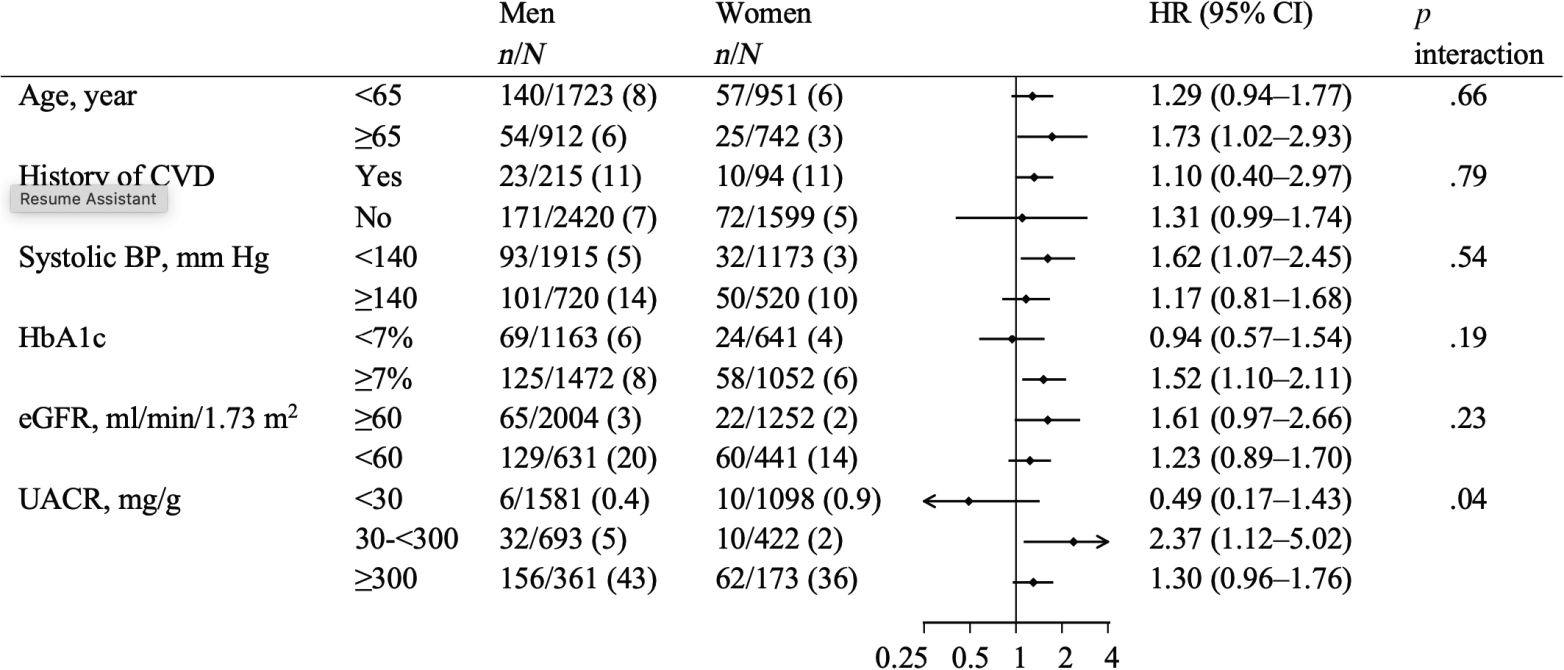
Association between sex (men versus women) and ESKD by baseline participant characteristics. Adjusted for age, history of cardiovascular disease, systolic BP, diastolic BP, HbA_1c_, eGFR, and log‐transformed UACR and stratified by institutions. BP, blood pressure; CI, confidence interval; CVD, cardiovascular disease; eGFR, estimated glomerular filtration ratio; HbA_1c_, glycated hemoglobin; HR, hazard ratio; UACR, urine albumin‐to‐creatinine ratio.

**FIGURE 4:**
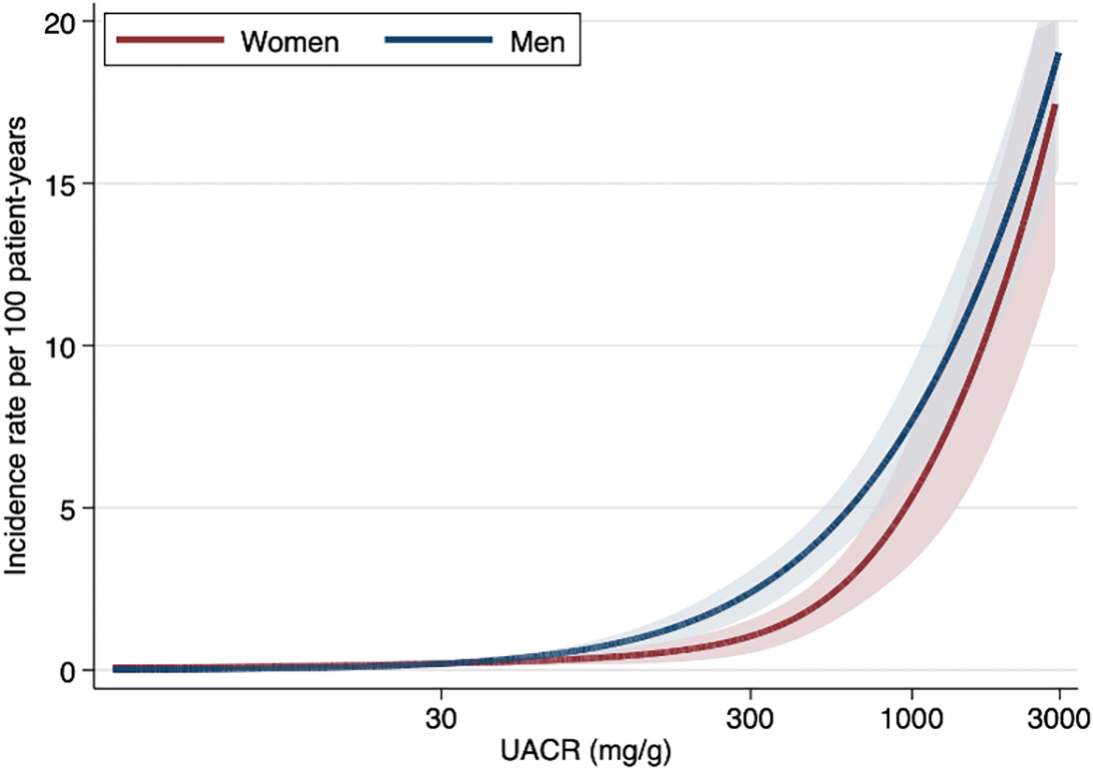
Incidence rates of ESKD according to baseline UACR by sex. Adjusted for age, history of CVD, systolic BP, diastolic BP, HbA_1c_, and eGFR. BP, blood pressure; CVD, cardiovascular disease; eGFR, estimated glomerular filtration ratio; ESKD, end‐stage kidney disease; HbA_1c_, glycated hemoglobin; UACR, urine albumin‐to‐creatinine ratio.

No differences were observed in the association of sex and ESKD across baseline participant subgroups (*p* for interaction >.19) except for UACR (Figure 3). Among patients with a UACR of 30–300 mg/g, men had a higher risk of ESKD than women (HR 2.37; 95% CI 1.12–5.02), compared to patient groups with UACR of <30 and ≥300 mg/g (*p* for interaction .04). A similar finding for UACR was detected after including death as a competing risk (Table S3). Incidence rates per 100 patient‐years were higher in men than women with a UACR of approximately 300 mg/g (Figure 4). No difference was observed in the incidence rates of ESKD between sexes across age at baseline (Figure S3). There were no differences in the relationship between sex and death across patient characteristics including UACR (*p* for interaction >.08) (Table S4).

### 3.4 eGFR and UACR trajectories by sex

The mean eGFR gradually decreased during follow‐up in both men and women, and no differences in mean eGFR were observed between sex (0.09 mL min^−1^ 1.73 m^−2^ year^−1^ higher in men versus women, 95% CI −0.09 to 0.28; *p* = .33) (Figure S4A). Geometric mean UACR slowly increased over time among both men and women and was 17% higher in men versus women (95% CI 6.3%–24%; *p* = .002 in men compared with women) (Figure S4B).

For the association of sex with clinical phenotypes of DKD, men were more likely to have progression of albuminuria (HR 1.18; 95% CI 1.02–1.37; *p* = .03) and a lower likelihood of regression of albuminuria (HR 0.69; 95% CI 0.52–0.93; *p* = .01) versus women (Table S5). In contrast, no associations were observed between sex and a rapid eGFR decline of >5 mL min^−1^ 1.73 m^−2^ year^−1^ (HR 0.93; 95% CI 0.65–1.35; *p* = .72).

## 4 DISCUSSION

Among patients with type 2 diabetes, men had significantly increased risks of ESKD and death versus women, even after adjustment for previously known risk factors such as HbA_1c_, eGFR, and albuminuria. Additionally, the sex difference in ESKD risk was prominent among patients with moderately increased albuminuria. In particular, men were associated with a higher risk of progression of albuminuria and a lower likelihood of regression of albuminuria compared with women. On the other hand, no difference was found between sex and eGFR trajectories. These findings may have important implications for improving risk stratification and preventive strategies for kidney disease progression in patients with type 2 diabetes.

We found that men had a higher risk of DKD progression compared to women with type 2 diabetes, which is mostly consistent with previous reports.^4^, ^5^, ^9^ In prospective studies of CKD population, men had a higher risk of ESKD and death both with and without adjustment for the presence of diabetes.^4^, ^9^ A prospective study of type 2 diabetes also indicated that male sex was an independent risk factor for a steep eGFR decline of >3.5 mL min^−1^ 1.73 m^−2^ year^−1^.^5^ Additionally, several cross‐sectional studies of type 2 diabetes found that men had a higher prevalence of albuminuria than women.^10^, ^11^ These findings were consistent with data from the Japanese Society of Dialysis Therapy registry, demonstrating substantially higher incidence rates of renal replacement therapy due to diabetic nephropathy in men than in women.^12^ In contrast, a retrospective study of type 2 diabetes in Japan has reported conflicting findings that females had a greater eGFR decline versus males (−3.5% ± 2.7% vs. −2.0% ± 2.2% per year).^13^ The association of sex with the risk of kidney outcome has been limited to type 2 diabetes only, but the current study was notable for evaluating ESKD in a large population with type 2 diabetes while adjusting for risk factors of kidney disease progression.

This study found a substantial increase in ESKD risk in men versus women with microalbuminuria at baseline. This may be partly explained by our findings that men were more likely to demonstrate an increase in albuminuria and were less likely to have a reduction in albuminuria compared with women during follow‐up. A previous prospective study of type 2 diabetes has similarly reported that men had an increased incidence of microalbuminuria compared to women.^14^ Changes in albuminuria have been widely recognized as reliable surrogate endpoints for kidney prognosis beyond one‐point albuminuria measurement,^15^, ^16^ which may have led to a worse kidney outcome in men than women in the current cohort. Another cohort study also suggested that the higher rates of obesity and smoking in men versus women can promote the development of proteinuria.^17^ Moreover, because albuminuria is indicative of microvascular dysfunction, the sex‐albuminuria interaction may reflect the presence of sex differences in the pathophysiology of DKD.^18^, ^19^ A prospective cohort study of adolescents with type 2 diabetes reported that females had a threefold greater risk of developing hyperfiltration over 5 years compared to males.^20^ In short, our findings suggest that sex differences may exist in the impact of albuminuria on the progression of DKD, although further evaluation is needed to elucidate its underlying mechanism.

In addition to the sex‐albuminuria interaction, there are several possible explanations for the association of sex and kidney prognosis in type 2 diabetes. First, in a meta‐analysis of the general public and CKD patients, males had a higher risk of acute kidney injury compared to females.^21^ Acute kidney injury is also frequently seen in patients with diabetes and is a well‐known risk factor for the development of CKD and kidney failure,^22^ which may lead to an increased incidence of ESKD in men compared to women. Second, in a pooled analysis of six cohorts, hypertension was a stronger risk factor for CKD progression and ESKD in men than women.^23^ However, in this study, risk modification was not observed according to BP levels at baseline. Lastly, gender‐related behavior, specifically regarding disease management and therapeutic strategies, may be involved in the sex‐based differences in kidney prognosis.^24^

We observed no sex differences in eGFR trajectories over time, although the annual eGFR slope is a useful surrogate end point for kidney prognosis in recent clinical trials.^16^ This observation can be explained by various reasons. Diabetes is a risk factor for acute kidney injury requiring dialysis^25^; however, this study collected eGFR data on a yearly basis, which might have resulted in missed cases of acute kidney injury, leading to dialysis. Diabetes is also associated with the early initiation of dialysis in patients with high eGFR levels,^26^ which may have partly resulted in similar eGFR values between the sexes. In addition, the timing of dialysis initiation is not based on eGFR alone, and other clinical manifestations, including electrolyte acid–base metabolism disorders, volume overload, and heart failure, may possibly require dialysis, regardless of eGFR.^27^

Sex hormones can directly and indirectly mediate hemodynamics and inflammation in the kidney. The influence of sex hormones on the renin‐angiotensin system (RAS) may be related to response to RAS blockade, leading to the sex‐based differences in DKD progression.^28^ Testosterone levels may also affect the development of hypertension and hypertension‐induced kidney injury in diabetes.^29^ For the detailed mechanisms underlying the association between sex hormones and DKD progression, recent studies have demonstrated that both sex and sex hormones affect the expression of transforming growth factor‐β1, which can induce kidney injury.^30^ Furthermore, some studies have reported that kidney complication manifests more than 10 years later in women than in men,^31^ suggesting the presence of protective effects of estrogen on the kidney during premenopausal period.^32^, ^33^ However, the current study did not find any difference in the association between sex and kidney outcomes across age.

The current study also demonstrated the sex differences in the association with mortality in type 2 diabetes, which was inconsistent with previous studies. Previous meta‐analyses have reported that women with diabetes showed a greater risk of all‐cause and cardiovascular mortality compared to men, although there was significant heterogeneity between studies.^34^, ^35^ This may be because of the variations in the prevalence of cardiovascular risk factors across sex in type 2 diabetes.

Our study's strengths include real‐world clinical data with a large sample size and long follow‐up duration. However, there are several limitations. Although we adjusted for multiple risk factors, we cannot exclude the effects of residual confounding factors such as body mass index, smoking status, and medications (eg, hyperglycemic and hypertensive agents). Moreover, sex‐specific factors such as pregnancy, menopause, and hormone replacement were not recorded in this cohort, and thus we cannot consider the impact of those factors on sex differences in DKD progression. Underlying mechanisms for the sex differences require to be elucidated. In addition, variations between centers regarding the requirement and processes for obtaining informed patient consent and enrolling patients can also lead to selection bias.

In conclusion, men have a higher risk of ESKD compared to women with type 2 diabetes, suggesting that sex differences may affect the risk of kidney disease progression due to type 2 diabetes. Early detection and management of modifiable risk factors and other comorbidities may slow the progression of DKD and prevent kidney failure in patients with type 2 diabetes. Further studies are needed to elucidate the mechanisms behind these sex differences.

## FUNDING INFORMATION

This research did not receive any specific grant from funding agencies in the public, commercial, or not‐for‐profit sectors.

## CONFLICT OF INTEREST STATEMENT

There are no conflicts of interest to declare.

## Supporting information


**Data S1.** Supporting Information
