# Supplementary material for: Sex differences in risk factors for end‐stage kidney disease and death in type 2 diabetes: A retrospective cohort study
Source: J Diabetes. 2023 Feb 13;15(3):246–54. doi: 10.1111/1753-0407.13367 (PMC10036257; doi:10.1111/1753-0407.13367)
Supplement: Supplementary file 1 — Data S1. Supporting Information [file JDB-15-246-s001.pdf]

Supplementary Figure 1: Hazard ratios for the association between baseline covariates and death

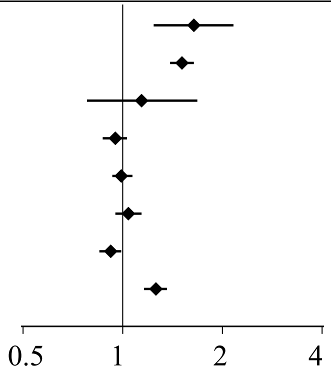

	Hazard ratio (95% CI)	P value
Men (versus women)	1.64 (1.24–2.16)	0.001
Age (+5 year)	1.51 (1.39–1.64)	<0.001
History of CVD (vs. no history)	1.14 (0.78–1.68)	0.50
Systolic BP (+10 mmHg)	0.95 (0.87–1.03)	0.20
Diastolic BP (+5 mmHg)	0.99 (0.93–1.07)	0.89
HbA1c (+1%)	1.04 (0.95–1.14)	0.35
eGFR (+10 ml/min/1.73 m^2)	0.92 (0.85–0.99)	0.02
UACR (+log 1 mg/g)	1.26 (1.16–1.36)	<0.001

Supplementary Figure 2: Kaplan–Meier curves for death in men and women

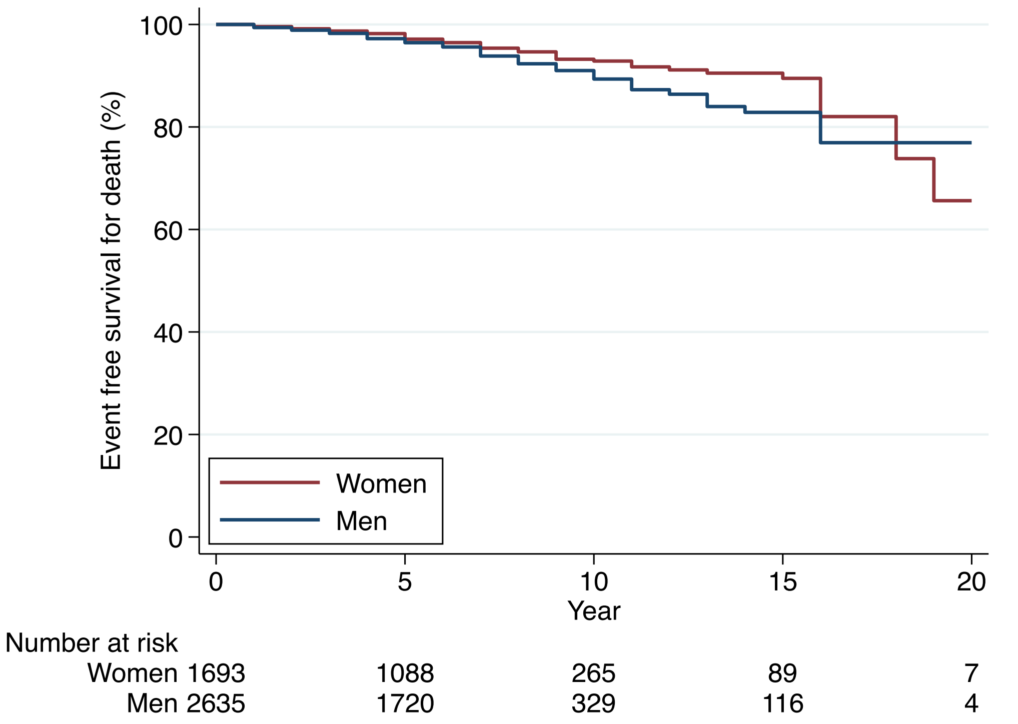

Supplementary Figure 3: Incidence rates of ESKD according to baseline age by sex

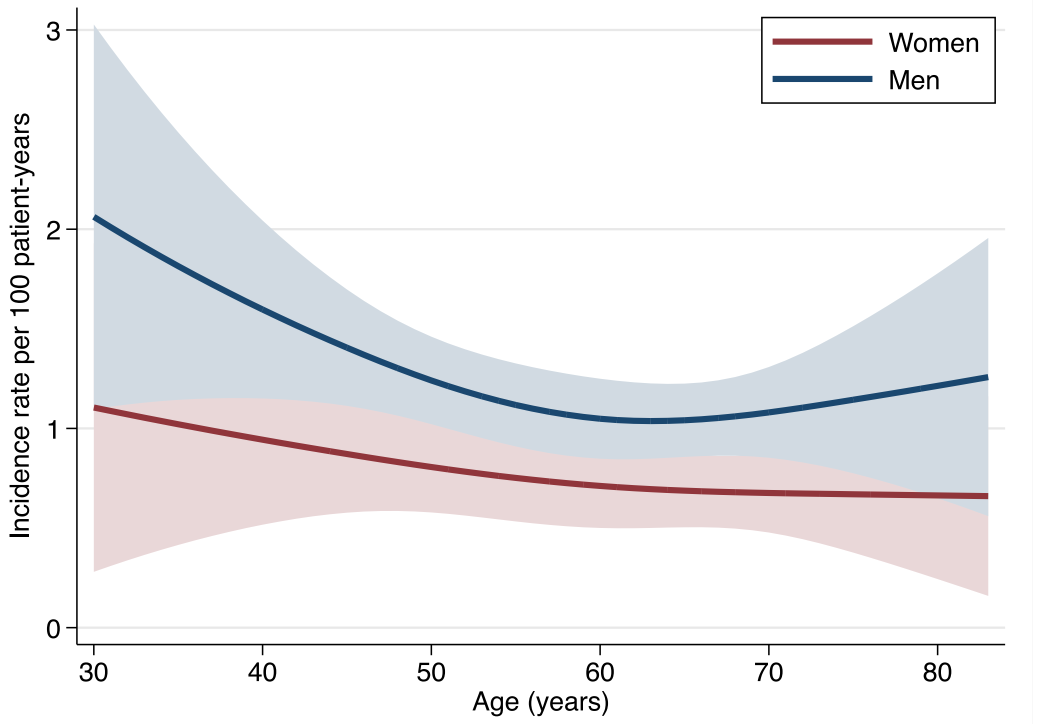

Adjusted for history of cardiovascular disease, systolic BP, diastolic BP, HbA1c, eGFR, and log-transformed UACR.

Supplementary Figure 4: Trajectories of eGFR (A) and UACR (B) by sex during follow-up

A

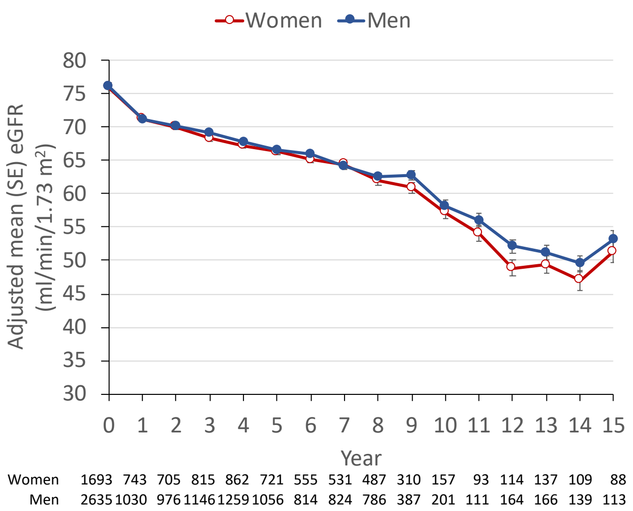

B

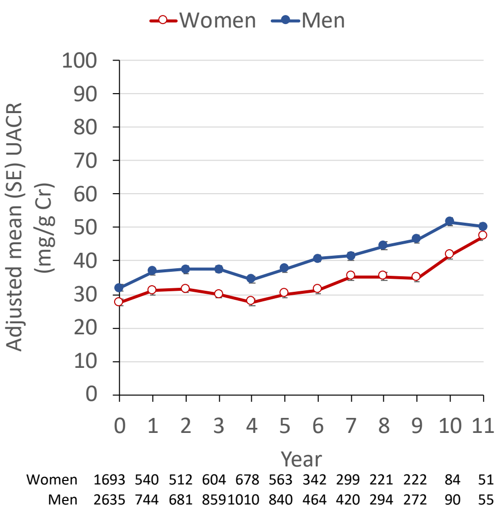

eGFR, estimated glomerular filtration ratio; UACR, urine albumin-to-creatinine ratio.

Supplementary Table 1: Numbers of men and women by year of registration

Year of registration	Men, n (%)	Women, n (%)	Total, n
1985–1989	9 (50)	9 (50)	18
1990–1994	137 (55)	111 (45)	248
1995–1999	8 (57)	6 (43)	14
2000–2004	1301 (62)	797 (38)	2098
2005–2009	834 (63)	494 (37)	1328
2009–2011	18 (55)	15 (45)	33
Total	2307 (62)	1432 (38)	3739

Among all patients (n=4328), the year of registration was recorded for 3739 patients (86.4%).

Supplementary Table 2: Hazard ratios for the association between baseline covariates and ESKD including death as a competing risk

	Hazard ratio (95% CI)	P value
Men (versus women)	1.41 (1.04–1.90)	0.03
Age (+5 year)	0.85 (0.79–0.91)	<0.001
History of CVD (vs. no history)	1.17 (0.75–1.82)	0.50
Systolic BP (+10 mmHg)	1.02 (0.94–1.09)	0.67
Diastolic BP (+5 mmHg)	1.01 (0.96–1.07)	0.74
HbA1c (+1%)	1.16 (1.08–1.24)	<0.001
eGFR (+10 ml/min/1.73 m^2)	0.74 (0.69–0.81)	<0.001
UACR (+log 1 mg/g)	2.12 (1.94–2.31)	<0.001

Adjusted for age, history of CVD, systolic BP, diastolic BP, HbA1c, eGFR, and log-transformed UACR and stratified by institutions.
CVD, cardiovascular disease; BP, blood pressure; eGFR, estimated glomerular filtration ratio; UACR, urine albumin-to-creatinine ratio.

Supplementary Table 3: Association between sex (men versus women) and ESKD by baseline patient characteristics including death as a competing risk

		HR (95% CI)	P interaction
Age, year	<65	1.24 (0.88–1.73)	0.25
	≥65	2.06 (1.11–3.83)	
History of CVD	Yes	1.37 (0.57–3.27)	0.35
	No	1.44 (1.05–1.98)	
Systolic BP, mmHg	<140	1.38 (0.88–2.17)	0.42
	≥140	1.45 (0.97–2.16)	
HbA1c	<7%	1.14 (0.68–1.90)	0.53
	≥7%	1.58 (1.10–2.26)	
eGFR, ml/min/1.73 m^2	≥60	1.52 (0.93–2.49)	0.51
	<60	1.34 (0.93–1.94)	
UACR, mg/g	<30	0.28 (0.08–1.04)	0.01
	30–<300	2.41 (1.04–5.54)	
	≥300	1.37 (0.99–1.89)	

Adjusted for age, history of cardiovascular disease, systolic BP, diastolic BP, HbA1c, eGFR, and log-transformed UACR and stratified by institutions.

Supplementary Table 4: Association between sex (men versus women) and death by baseline patient characteristics

		Men n/N (%)	Women n/N (%)	HR (95% CI)	P interaction
Age, year	<65	63/1723 (4)	20/951 (2)	1.94 (1.15–3.26)	0.31
	≥65	101/912 (11)	57/742 (8)	1.57 (1.12–2.19)	
History of CVD	Yes	25/215 (12)	8/94 (9)	2.16 (0.88–5.32)	0.64
	No	139/2420 (6)	69/1599 (4)	1.58 (1.18–2.13)	
Systolic BP, mmHg	<140	102/1915 (5)	41/1173 (4)	1.77 (1.22–2.58)	0.82
	≥140	62/720 (9)	36/520 (7)	1.51 (0.99–2.33)	
HbA1c	<7%	73/1163 (6)	27/641 (4)	1.69 (1.07–2.66)	0.60
	≥7%	91/1472 (6)	50/1052 (5)	1.59 (1.11–2.27)	
eGFR, ml/min/1.73 m^2	≥60	81/2004 (4)	38/1252 (3)	1.56 (1.04–2.32)	0.41
	<60	83/631 (13)	39/441 (9)	1.98 (1.32–2.96)	
UACR, mg/g	<30	71/1581 (4)	31/1098 (3)	1.87 (1.22–2.89)	0.08
	30-<300	48/693 (7)	20/422 (5)	1.93 (1.12–3.32)	
	≥300	45/361 (12)	26/173 (15)	0.96 (057–1.61)	

Adjusted for age, history of cardiovascular disease, systolic BP, diastolic BP, HbA1c, eGFR, and log-transformed UACR and stratified by institutions.

Supplementary Table 5: Association between sex (men versus women) and DKD phenotypes

	Men<br>n/N (%)	Women<br>n/N (%)	HR (95% CI)	P value
Progression of albuminuria	461/1993 (23)	304/1333 (23)	1.18 (1.02–1.37)	0.03
Regression of albuminuria	113/679 (17)	85/359 (24)	0.69 (0.52–0.93)	0.01
	Men<br>n/N (%)	Women<br>n/N (%)	OR (95% CI)	P value
Rapid eGFR decline	100/2384 (4)	60/1539 (4)	0.93 (0.65–1.35)	0.72

Adjusted for age, sex, history of cardiovascular disease, systolic BP, diastolic BP, HbA1c, eGFR, and log-transformed UACR and stratified by institutions.

eGFR, estimated glomerular filtration ratio; UACR, urine albumin-to-creatinine ratio.
